# A qualitative descriptive study of the contextual factors influencing the practice of emergency nurses in managing emerging infectious diseases

**DOI:** 10.1080/17482631.2019.1626179

**Published:** 2019-06-11

**Authors:** Stanley K. K. Lam, Enid W. Y. Kwong, Maria S. Y. Hung, Samantha M. C. Pang, Wai T. Chien

**Affiliations:** a School of Nursing, Tung Wah College, Kowloon, Hong Kong; b School of Nursing, Hong Kong Polytechnic University, Kowloon, Hong Kong; c The Nethersole School of Nursing, The Chinese University of Hong Kong, New Territories, Hong Kong

**Keywords:** Emerging infectious diseases, epidemics, emergency nurses, nursing, qualitative study

## Abstract

**Purpose**: Emergency nurses are engaged in the management of epidemic events that unfold along with the evolution of diseases. The goal of this study was to explore the contextual factors that inhibited the ability of emergency nurses to perform their duties in response to an outbreak.

**Methods**: A qualitative descriptive design was used to explore the experiences and perceptions of emergency nurses. Participants were purposively recruited from 12 emergency departments in Hong Kong. Semi-structured face-to-face individual interviews were conducted with 26 emergency nurses. The audio-recorded interviews were transcribed verbatim and interpreted with a thematic analysis approach.

**Results**: Four intertwined themes emerged from the analysis: resource constraints, threats of infection, ubiquitous changes and lingering uncertainties. These themes portrayed the constraints and challenges surrounding the work environment of emergency nurses.

**Conclusion**: This study described the instabilities and vulnerabilities of the circumstances in which the emergency nurses were situated in during epidemic events. The findings shed light on the importance of hospitals and emergency departments in addressing both the technical problems and adaptive challenges that face emergency nurses during epidemic events.

## Introduction

Despite efforts to promote disease surveillance and infection control, the outbreak of emerging infectious diseases (EIDs) has remained a major threat to global public health and presents enormous challenges for healthcare systems worldwide (Weber, Rutala, Fischer, Kanamori, & Sickbert-Bennett, 2016). During the course of an EID event, accident and emergency departments (AEDs) take on a wider role by actively participating in the planning for and response to public health threats from EIDs on top of continuing to manage urgent illnesses and injuries. Apart from the distinguishing feature of emergency care in offering prompt intervention to patients in critical condition, the public health function of AEDs in assessing, monitoring and promoting the health of community members is considered a longstanding priority in managing epidemics (Menchine, Zhou, Lotfipour, & Chakravarthy, 2016). The extended nature of emergency services in an EID event requires emergency nurses to demonstrate not only the capability to offer emergency care services to the public but also the capacity to participate in public health responses and combat a large-scale public health emergency.

In addition to their important role in the public health response to an epidemic event, emergency nurses also face barriers to fulfilling their duties in the course of EID management. They have been shown to be non-compliant with guidelines and protocols intended to prevent the spread of EIDs, such as adherence to hand hygiene practices (Muller, Carter, Siddiqui, & Larson, 2015) and personal protective equipment (PPE) usage (Baduge, Moss, & Morphet, 2017). Studies have revealed that limitations in time and resources were the major cause of non-compliance with recommended practices (Lam, Kwong, Hung, & Pang, 2016; Muller et al., 2015). Interestingly, it has also been reported that the issue of emergency nurses’ non-compliance with guidelines could still occur, regardless of the availability of sufficient resources (Lam & Hung, 2013). This suggests the existence of other contextual factors that affect the decision about whether to conform to a protocol. Studies have also been conducted to identify factors affecting the practices of healthcare workers in the course of outbreak management. These studies showcase the important role of institutions in offering training, guidance and incentives to frontline staff (Craig et al., 2018; Greenberg et al., 2019). What is less clear is which factors in the emergency care setting adversely affect the ability of emergency nurses to engage in an EID outbreak response. This lack of knowledge could lead to difficulties in devising appropriate strategies to address non-compliance and could directly affect the role of emergency nurses in EID management. Understanding emergency nurses’ experiences and perceptions of disease management during epidemic events might help identify and address the barriers to their involvement in EID management. The goal of this study was to identify and explore the contextual factors that inhibited the ability of emergency nurses to perform outbreak-response-related duties according to established protocols (Lam, Kwong, Hung, & Pang, 2016; Muller et al., 2015).

## Methods

### Design

The present study adopted a qualitative descriptive design. A qualitative descriptive design uses a naturalistic perspective to understand a phenomenon in a natural setting and emphasises the exploration of ascribed meaning from individuals who are involved (Bradshaw, Atkinson, & Doody, 2017). Such an approach can offer a rich description of a phenomenon about which little is known (Colorafi & Evans, 2016). The goal of a qualitative descriptive study is to obtain knowledge of the experiences, events and interactions of a phenomenon from the viewpoint of the insiders (Bradshaw et al., 2017). Because the goal of this study was to identify influential contextual factors that hindered the ability of emergency nurses to perform outbreak-response-related duties, a qualitative descriptive design was particularly relevant.

### Participants and eligibility

A purposive sampling strategy was used to recruit 26 participants from 12 accident and emergency departments in Hong Kong. Such a sampling strategy allows researchers to identify information-rich individuals within the phenomenon of interest by virtue of their knowledge and experience (Etikan, Musa, & Alkassim, 2016). This strategy can facilitate an in-depth understanding of the research area (Yin, 2011). The eligibility criteria in the selection of participants were identified as follows: emergency nurses who (1) worked on a full-time basis in Hong Kong; (2) were able to understand and communicate in Cantonese; and (3) were willing to share their experiences in the management of EIDs. Part-time emergency nurses were excluded. Sampling continued until data saturation was achieved. A total of 26 emergency nurses from 12 AEDs in Hong Kong participated in the study.

### Data collection

The first author (SKKL) conducted semi-structured, face-to-face individual interviews to solicit the participants’ experiences and perceptions of the challenges and constraints encountered in the course of EID management. An interview agenda, which consisted of open-ended questions, was utilised in the interviews (Box 1). Using an interview agenda can offer direction to the participants regarding the inquiry of the study while maintaining the openness and flexibility of the participants’ description of their experience as much as possible (Sandelowski, 2000). To facilitate the subsequent data analysis, the interviews were audiotaped with the participants’ consent and permission. The interviews lasted 45–150 min.

**Box 1.** The interview agenda.

Please describe the factors in the emergency practice environment that inhibited your engagement in EID management. Please explain some of your experiences in which your works and practices in EID management were inhibited by these factors. In what way did these factors inhibit your works and practices?

### Data analysis

The interview tapes were transcribed verbatim for data analysis. All of the transcripts were checked against the original recordings to ensure the exactness of the transcription. A thematic analysis strategy was adopted to guide the process of data analysis. This strategy is a practical and useful approach for qualitative analysis that aims at identifying and integrating patterns within the data (Braun & Clarke, 2006). The data analysis consequently generated four overarching themes from the information.

### Ethical considerations

Prior to commencement of the study, ethical approval was granted by the Human Ethics Committee of the Hong Kong Polytechnic University (no reference number, approved November 2013). The anonymity and confidentiality of the participants were ensured and maintained throughout this study. During recruitment, potential participants were provided with verbal and written information regarding the nature of the study and their participation. They were also informed that their involvement in the study was voluntary. Written informed consent was obtained from all participants.

## Results

The participants’ descriptions were used to identify the contextual factors that inhibited their ability to perform outbreak-response-related duties. The factors were multifaceted and profoundly impacted the daily practice of emergency nurses. These were (1) resource constraints, (2) threats of infection, (3) ubiquitous changes and (4) lingering uncertainties.

### Resource constraints

A recurring theme in the interviews was a sense among the participants that their workload drastically increased with the emergence of an EID. All of the participants reported that EID management created an additional workload on top of their already heavy workload. The source of the increased workload was mainly the increased patient attendance in the AED. A participant described her understanding of the reasons behind the increased patient attendance as follows:

‘While there was an announcement of an EID or there was an outbreak of a new disease, patient attendance would suddenly increase. People just rushed to the AED for a check-up, for fear they had contracted the new disease. They were so nervous that many of them just showed up with very mild symptoms.’ (P17)

The problem of insufficient facilities to serve the needs of suspected infectious patients was also frequently cited by participants as an EID management issue. In addition to insufficient isolation rooms in the wards to meet patient admission requirements, the isolation facility within the AED was frequently described by the participants as inadequate. A nurse who worked in a hospital revealed that the isolation facility within the AED where she worked was so limited that the only room with an isolation facility was the resuscitation room:

‘The area of our department is not very large, and there is only one installed negative pressure room, which is the resuscitation room. If there is an infectious patient occupying the room, we would have less resuscitation room for patients in critical condition. Sometimes we must share the negative pressure room by dividing it into several partitions with sliding doors.’ (P10)

In addition to the isolation facilities, short staffing was identified as another major resource constraint facing emergency nurses in the management of EIDs. In fact, human resource shortages were constant issue for the healthcare service providers. The workload from managing EIDs created additional pressure on an already tight human resource supply. Discussing the staffing shortage issue, a participant said,

‘Manpower in the department is already tight, and all of us were already exhausted. While there was an upsurge in patient attendance during an EID event, sometimes we only had one staff member to handle the responsibility of two. Not to mention the situations when there are staff who take sick leave.’ (P13)

Another nurse also mentioned that staff taking sick leave during an EID event created a “vicious circle” that further depleted the department’s human resources for emergency nurses managing EIDs:

‘When there was insufficient staff and increased patient numbers, the workload of every nurse drastically increased. This might be harmful to staff health, as the workload could overload our capacity, both physically and psychologically. The staff become exhausted and burned out. This is how staff members get sick and call in sick.’ (P3)

### Threats of infection

The majority of the participants perceived that engagement in EID management entailed a significant health risk to the frontline emergency nurses. They emphasised that the risk of infection for emergency nurses was comparatively higher than for those working in other departments, which adversely affected their willingness to engage in an EID outbreak response. The participants indicated that the AED was the “first line of the frontline” in confronting an EID, which induced a substantial risk of infection among departmental staff. The following pertinent remark was made by a participant describing his experience during the Severe Acute Respiratory Syndrome (SARS) outbreak:

‘We were even unable to be sure of our own safety. When there was an outbreak, the accident and emergency department would be responsible for the first line of defence, making us subject to the risk of infection. We were terrified and worried about our own health.’ (P3)

In addition to their own personal health, the health of their significant others was also a concern for emergency nurses when participating in EID management. They mentioned they might become a “source of infection” and transmit pathogens to family members with whom they were in daily contact. A nurse described these concerns as follows:

‘If I am infected while handling patients who are infected, I would become the source of infection and bring pathogens to my family. I don’t want to be infected, and I don’t want to infect my family.’ (P1)

Among the nurses who identified an elevated risk of infection, most stated that their department and hospital had implemented various strategies to protect them from infection, such as providing PPE and vaccination. However, the participants doubted the effectiveness of these measures. For example, a nurse expressed that she was not very confident about the effectiveness of PPE in protecting her from the Ebola virus:

‘I cannot be sure if I am 100 per cent protected from Ebola infection with the PPE we are provided. In the news reports, healthcare workers in other countries would have PPE that looks like a spaceman suit that seems to provide better protection, but what we are provided is far less sophisticated.’ (P18)

In addition, a nurse stated her reasons for refusing to be vaccinated, even as she was required to participate in managing EIDs. She had never received any vaccinations before, and her opinion was based on her perception:

‘First, to my knowledge, there is no vaccine available that is effective at protecting us from a new infectious disease. Second, people could become infected and get sick even if they were vaccinated, so I don’t see the point of getting vaccinated. Third, the side effects of vaccination are quite intense, and I don’t want to go through that when I am healthy and feeling alright.’ (P9)

### Ubiquitous changes

The work situations of emergency nurses were subject to abrupt changes in association with the progression of an EID, and these changes constituted barriers to disease management. The most frequently encountered change was an unstable disease situation. While multiple types of EIDs might appear all at once, the participants described how the emphasis on disease management constantly changed depending on the dominating disease at a particular period of time. These changes caused the nurses to experience difficulties in orientation towards the respective circumstances. Confronted with threats from multiple diseases, a participant shared her experience managing the Ebola virus in the presence of other types of EIDs:

‘Different kinds of newly recognised infectious diseases come at once, and the disease situation changes frequently. Not long ago, the “new SARS” (Middle East respiratory syndrome) had dominated, but then it changed to the avian flu. Most recently, the Ebola virus has been severe.’ (P19)

The provision of information was an important source of guidance for emergency nurses to identify their work duties and practices during the course of EID management. Commenting about the dissemination of EID information, a nursing officer summed up her experience in disease management with the following remark:

‘All along in the management of different kinds of EID, the information we received from the department changed too rapidly, almost every day. Information about the disease is largely indefinite and incomplete.’ (P6)

As guidelines and practices mainly focus on disease management, targeting disease surveillance and infection control, practices were changed and implemented according to the type of prevalent EID and the progression of the outbreak. More stringent infection control guidelines were established to tie in with elevated incidences of EID infection and prevent nosocomial infections. Although the majority of participants valued the relevance of these accelerated infection control measures in protecting healthcare personnel and patients against EIDs, the changes to the required measures and procedures when handling patients with suspected infection status were frequent and led to difficulties in adoption and execution. The difficulty, as described by a nurse, was that their adjustments could hardly meet the amendments:

‘There are usually no major changes in practice, but only subtle changes, which are so frequent and rapid that when I am in the middle of adjusting to the changes, the situation has already changed.’ (P10)

Most of the participants agreed that changes were necessary for effective disease management. However, some participants with higher rankings indicated that there were difficulties in promoting the new measures in practice because of a reluctance among some of their nursing colleagues to accept changes. An advanced practice nurse commented,

‘Once there are changes in the existing guidelines, no matter whether it’s new infection control measures or isolation policies … some staff would hold onto their existing practices and beliefs about those guidelines … which caused difficulties in implementing the changes.’ (P21)

### Lingering uncertainties

The uncertainty surrounding a disease situation was another contextual factor that affected emergency nurses’ performance of outbreak-response-related duties. The participants described the emergency care environment to be inherently embedded with uncertainty, casting significant doubt on how they responded to an impending infectious disease emergency. For example, a junior nurse said,

‘One can never predict what types of patients will appear. As their infectious status remains unknown, we are unsure about the actions that should be taken for them.’ (P9)

Another emergency nurse echoed a similar opinion on the uncertainties concerning the infectious status of patients, stating that she was not only uncertain about patients’ infectious status, but that she would be infected likely without even being able to identify the origin of the infection:

‘I am in contact with many different patients a day, and if I found myself infected with the new disease (EID), I would wonder who was the one who infected me. This actually happens in practice. The pathogens cannot be seen or touched, one would not be able to tell if one is being infected if there are no symptoms unless you go for a check-up.’ (P17)

The issue of uncertainty concerns junior and senior nurses in the emergency department. A participant with over 20 years of experience in emergency nursing reported the following thoughts about the unpredictable nature of the work environment:

‘There would be no fixed routines in our practice, as the events we face would never be fixed. Peculiar situations just come all of a sudden, and all we can do is improvise, play it by ear.’ (P16)

Uncertainty also originated from a lack of understanding and clarity about EID situations. The majority of the participants mentioned that information on emerging and unforeseen diseases, particularly at the early stages of an outbreak, was ambiguous and confusing. A participant reported his experience from a previous SARS and H1N1 pandemic, in which information about the newly recognised diseases was grounded in “nothing more than hearsay”. Another participant described a similar experience, indicating that the manifestation of uncertainty was from confusion over information about the disease. The nurse remarked,

‘The disease information about newly-identified EIDs seems to be delayed and incomplete. It is not standardised across departments, adding confusion to the situation.’ (P14)

## Discussion

The findings depict nurses’ collective consciousness regarding the instabilities and vulnerabilities of their circumstances during an epidemic event and uncovered possible contextual barriers to the ability of emergency nurses to perform outbreak-response-related duties according to established protocols for combating EIDs. The major barriers facing nurses under such circumstances were mainly infection risk, resource shortages, workplace changes and uncertainties surrounding the situation. These impediments in the everyday practices of emergency care delivery were the fundamental sources making up the tasks and issues facing emergency nurses and that compelled them to engage in EID management.

The challenging situations described by the emergency nurses were largely in line with those outlined in previous studies on nurses from other care settings. These studies have highlighted the various challenging issues facing nurses during EID outbreaks (Kim, 2018; McMullan, Brown, & O’Sullivan, 2016). The identified issues often pertained to workplace susceptibility and resource constraints, which nurses are used to encountering and handling in their everyday practice. Furthermore, the changes and uncertainties in the emergency care setting during an epidemic worsen those issues and create unique challenges and obstacles to nursing practices. Indeed, change and uncertainty, which are inherent to the nature of the healthcare industry, are inevitable challenges facing nurses and other healthcare workers, and they create hesitation and fear and that affects the performance and quality of care delivery (Arrowsmith, Lau‐Walker, Norman, & Maben, 2016). During an epidemic event, the healthcare environment is often intertwined with urgency, uncertainty and change, and this scenario requires marked effort from workers to seamlessly maintain healthcare system operations (Cranley, Doran, Tourangeau, Kushniruk, & Nagle, 2012). Similar results have also been reported in the literature, highlighting the hardships and struggles from bouts of uncertainty and volatility that nurses must endure as the first-line responders under disastrous circumstances (Lam, Kwong, Hung, Pang, & Chiang, 2018). These findings suggest that nurses are situated in an unsettled circumstance during their participation in EID management.

The emergency nurses in this study reported encountering an unstable situation during EID management. They were unable to acquire sufficient clinical information to devise appropriate responses in EID management, which confounded their attempts to implement routine practices. As indicated by the findings, they developed a sense of anticipated crises in facing an EID event, which created considerable doubt and anxiety about their personal wellness and competence in managing an EID. On the other hand, this sense of crisis has also fostered nurses’ awareness of the severity of the disease situation, which enhanced their vigilance against EIDs. The literature suggests that when healthcare workers underestimate the severity of a public health event, it could result in laxness in handling any issues (Dionne, Desjardins, Lebeau, Messier, & Dascal, 2018). These findings shed light on the importance of maintaining an optimal level of risk perception and crisis awareness among nurses during an EID event to promote their alertness and prudence at work without triggering panic.

During the management of a public health event, assistance and support from hospital administration are some of the most important determinants of the preparedness and competence of the nursing workforce and workers in other healthcare disciplines. The findings show that nurses expected to encounter unanticipated challenges when responding to EID events, such as staffing shortages and inadequate facilities, and that the adverse effects on their practice can only be mitigated by institutional action. In the absence of adequate support and action from hospitals, the quality of healthcare delivery and nurses’ incentives and intentions to work could be hampered (Filice et al., 2013). Because the findings highlighted some major challenges and constraints emergency nurses encountered during epidemic events, hospitals should adapt and accentuate strategies for supporting nurses and other outbreak responders to better equip them to handle pressing needs on the frontline.

One of the main drawbacks indicated by the findings was the inadequacy of essential resources, such as human resources and isolation facilities, during past outbreaks. The challenging situations described by the emergency nurses in the findings are largely in line with those of previous studies on nurses from other care settings. To effectively optimise resource allocation during a public health emergency, resource triage can be implemented. Such a triage procedure could ensure the meticulous identification of priorities in maintaining core healthcare services. The cancelation or postponement of elective procedures could be another option for consideration. Through this approach of re-prioritising resource allocation, the surge capacity of hospitals during major EID events could be increased to meet expanded demand for healthcare services by the public. The standard of nursing practice could also be maintained with the sufficient provision of human resources and equipment.

## Conclusions

The emergency nurses’ experiences and perceptions of engaging in EID management encompassed a range of issues, and they collectively portrayed their engagement in EID management as adversely affected by resource constraints, threats of infection, ubiquitous changes and lingering uncertainties. These contextual barriers in the everyday practices of emergency care delivery were the fundamental antecedents that compelled emergency nurses to engage in suboptimal practices. The findings highlight on the importance of hospitals and emergency departments in addressing the technical problems and adaptive challenges facing emergency nurses amid epidemic events.
